# Emotional Trajectory at Different Career Stages: Two Excellent Teachers’ Stories

**DOI:** 10.3389/fpsyg.2020.01034

**Published:** 2020-06-09

**Authors:** Junjun Chen, John Chi-Kin Lee, Jihe Dong

**Affiliations:** ^1^Department of Education Policy and Leadership, The Education University of Hong Kong, Tai Po, Hong Kong; ^2^Department of Curriculum and Instruction, The Education University of Hong Kong, Tai Po, Hong Kong; ^3^School of Education, Shandong Women’s University, Jinan, China

**Keywords:** teacher emotion, teacher career stage, qualitative, emotional trajectory, China

## Abstract

The current study aimed to investigate excellent teachers’ emotional journey, particularly, the trajectory of emotional experiences and emotional labor strategies at different career phases. The research used a case-study approach to explore the storied experience of two teachers (female and male) who were bestowed the Provincial Excellent Teacher Award. They were close to retirement so they could retrieve emotional experiences from across their entire career. Individual semi-structured interviews were utilized as a major data source, supplemented with relevant documentation and phone calls to achieve data triangulation. Thematic analysis was adopted to deal with data. The findings demonstrated a dynamic pattern of emotions and emotional labor, transiting from one teacher career stage to another. It was observed that the female teacher experienced mixed emotions but the male teacher had more negative emotions at the early stage. Both teachers claimed more positive emotions in the middle stage and a high level of satisfaction in the late stage. Both of them employed genuine expression and surface acting strategies in the first two stages. In the late stage, the female teacher used a combination of genuine expression and deep acting with more empathy, whilst the male teacher adopted a combination of surface acting and genuine expression aiming for a neutral atmosphere. Social values, organizational demands professional self, and gender are discussed for possibly resulting in these discrepancies.

## Introduction

It has been a longstanding consensus that teaching is a high-risk profession (Capone and Petrillo, 2018; Taxer et al., 2019). Näring et al. (2011) claimed that “the work of teachers is being evaluated in more and more detail and this has also led to an increase in the emotional demands of teaching” (p. 12). It is especially true in the current crisis global wide as the new coronavirus is spreading and teacher professional life has been dramatically changed. A recent report released by the National Foundation for Educational Research (NFER, Worth and Van den Brande, 2019) in Britain has echoed that teachers’ occupational stress level has ratcheted up for the third consecutive year to the highest levels than ever. Teachers in China are not an exception. A recent meta-analysis project with 116 studies in China (Yang et al., 2019) has indicated that Chinese teachers have sustainably undergone a high level of negative emotions (e.g., stress, anxiety). The NFER report also warned that teachers’ negative emotions will multiply if there are no prompt and effective actions to provide teachers with more support. Hence, it is an urgent agenda to provide support to the teachers’ emotional side in order to make their teaching career more manageable and sustainable so that teachers can thrive, not just survive in their professional life (Mansfield et al., 2012) and so that we can also retain qualified teachers for promoting the quality of education (Bruland et al., 2017).

Classrooms and schools are flooded with emotions which highlights the significance of teacher emotions (Hargreaves, 2005; Day and Gu, 2013). Existing research has found that teacher emotions influence not only their instructional choices (Chen, 2019), well-being (Burić et al., 2019), and effectiveness (Huang et al., 2019b) but also their students’ emotions (Frenzel et al., 2018), the teacher – student relationship (Taxer et al., 2019), and learning outcomes (Burić, 2019). Given that teaching is full of emotions, how teachers manage their emotions becomes an important matter of interest. On a daily basis, teachers are experiencing and dealing with emotional demands from various aspects so they need to understand and competently manage their own and others’ emotions in order to be able to successfully deliver their teaching and complete other tasks (Cross and Hong, 2012). Furthermore, expressions of teachers’ emotional experiences are oftentimes guided by certain emotional-display rules, that is, there are distinctive implicit or explicit norms regarding the nature and intensity of emotions. In order to align their inner feelings and external expressions with emotions that are believed to be desirable or are prescribed in a given situation, teachers have to perform emotional labor (Burić, 2019).

Existing research has revealed a linkage between teachers’ emotions, emotion management, and effectiveness. For example, teachers with more positive emotions can devise better ways to handle their teaching to achieve better learning outcomes (Taxer and Frenzel, 2019). Furthermore, high-quality teachers manage their emotions effectively (Beltman and Poulton, 2019) and tend to achieve more teaching efficacy (Burić and Macuka, 2018) and well-being (Berkovich and Eyal, 2019). Therefore, if we could understand the trajectory of excellent teachers’ emotional experiences, it might provide helpful insights to other teachers to better manage their emotions, serve as a role model for their students, and achieve increased well-being and teaching effectiveness. The current study aims to investigate excellent teachers’ emotional journeys, particularly the trajectory of emotional experiences and emotional labor strategies at different career phases.

## Conceptual Framework for Teachers’ Emotional Experiences

### Teacher Emotions

Given that there is no consensual definition of emotion (Shuman and Scherer, 2014), this study agrees with Schutz et al. (2006) approach that regards emotions as “socially constructed, personally enacted ways of being that emerge from conscious and/or unconscious judgments regarding perceived successes at attaining goals or maintaining standards or beliefs during transactions as part of social-historical contexts” (p. 344). Farouk (2012) further defines teacher emotions as “internalized sensations that remain inert within the confines of their bodies but are integral to the ways in which they relate to and interact with their students, colleagues and parents” (p. 491). Teacher emotion is then considered to be “a part of a dynamic, continuously fluctuating system of meaningful experiences” (Zembylas, 2007, p. 61).

Scholars seem to agree on three characteristics of teacher emotions. First, according to teacher narrative studies, teachers experience different kinds of emotions. These seem to fall into two contrasting discrete kinds, such as positive and negative emotions, but with salient basic emotions identified. Frenzel et al. (2016) found that enjoyment is the most frequent positive emotion, while anger and anxiety are the most frequent negative emotions. A comparative study with Kindergarten teachers from China and Norway (Hong and Zhang, 2019) identified that positive emotions (e.g., enjoyment and happiness) are associated with children’s progress and performance, whereas negative emotions (e.g., anger and anxiety) relate to children’s behaviors and parent expectations. Likewise, Hong Kong primary teachers narrated that they enjoyed student trust and collegial support but experienced anger due to student misbehaviors (Wu and Chen, 2018). Gallant (2013) found that two primary teachers from Australia underwent guilt (alongside shame) and anger and love and passion when interacting with students and colleagues. Second, change in teachers’ emotional experience can occur at a certain time of period. Chen (2017) found that Chinese teachers experienced mixed emotions commencing with more negative emotions (e.g., worry and anxiety) and then more positive emotions (e.g., love and happiness) over a 6-year period with the same group of students. Third, teacher emotions have been identified to be sustainable. For example, two teacher leavers from United States reported sustainable negative emotions (e.g., sadness, anger, stress, and frustration) after reflecting on their teaching career and guilt after leaving teaching profession (Mawhinney and Rinke, 2018).

### Teachers’ Emotional Labor

Research highlights that teachers have not only passively experienced but also actively regulated their emotions, with the emotions teachers have experienced often differing significantly from those they have expressed to the teaching profession (Taxer and Frenzel, 2015; Wang et al., 2019). Hochschild (1983) defined emotional labor as “the management of feeling to create a publicly observable facial and bodily display” (p. 7) focusing on how individuals modify their emotional expressions from their truly experienced emotions for communicative purposes. Emotional labor encompasses three strategies, namely, surface acting, deep acting, and genuine expression (Hochschild, 1983; Diefendorff et al., 2005; Yin et al., 2019). Surface acting refers to when individuals externally express an emotion that differs from their experienced emotions without modifying their internal feelings, as evidenced by amplifying, hiding, faking, or suppressing an emotion. Deep acting occurs when teachers manage to internalize and modify an unwanted emotion to the extent that the emotion felt and the emotion expressed are more consistent. Genuine expression occurs when individuals experience emotions and spontaneously display them in a contextually appropriate manner.

Engaging in emotional labor strategies aiming at achieving positive learning and teaching outcomes is a constituent part of the teaching profession (Taxer and Frenzel, 2015). It is generally believed that teachers try to show positive emotions and avoid negative ones, as well as try to modify them for desirable consequences (Yin, 2016). Teachers sometimes have to suppress or hide their true feelings and express emotions that are fake but desirable or prescribed (surface acting) and/or try to actually feel the emotions that are expected to be expressed (deep acting) (Burić, 2019). For example, Gallant (2013) pointed out that teachers need to consciously regulate their emotions to establish an emotional balance especially with early year students. She found that both anger and care could be an emotional labor strategy as teacher’s anger may draw students’ attention and make them behave better. Moreover, it is also acceptable for teachers to naturally express their genuine emotions (e.g., enjoyment) in order to fulfill their teaching goals and expectations (Sutton et al., 2009). Nevertheless, Yin et al. (2019) commented that “regardless of how such strategies are conceptualized and explained, the expression of naturally felt emotions is seen as the most desirable, while deep acting is still more adaptive than surface acting in terms of outcomes” (p. 3).

### Teachers’ Emotions and Emotional Labor in Context

Neither emotion experienced nor emotional labor is an isolated individual process. Both are influenced by the context (Mesquita and Albert, 2007; Huang et al., 2019b). Kemper (1993) argued that the social structure may determine which emotions are inclined to be experienced and when, where, how, and why, and by whom they are expressed. Particularly, Sutton and Harper (2009) commented that the degree of emotional regulation is moderated by cultural norms. Western people prefer open expressions as they advocate self-assertion and independence, while eastern people tend to suppress or hide their emotions especially negative ones as they favor social harmony and interdependence. Yin (2016) further identified that teachers’ emotional labor in Chinese culture may be affected by ethical norms (e.g., collectivism). As aforementioned, a comparative study in China and Norway (Hong and Zhang, 2019) found a discrepancy between two groups of teachers in terms of negative emotions and emotional labor strategies. Chinese teachers tend to undertake more complex emotional labor strategies to encounter their negative emotions than their Norwegian counterparts do. Both surface acting and deep acting strategies were adopted by both groups; however, Norwegian teachers were more likely to adopt deep acting and take professionalism and goals as the internal motivation of their work. Likewise, Lee and Yin (2011) found that Chinese do not often express negative emotions in public arenas but try to use indirect ways to avoid open conflicts and achieve social harmony.

Furthermore, some social forces are influential on individuals’ emotion management (Akin et al., 2014). For example, gender has been a long-standing focus within the literature on emotion management and the teaching profession. Gendered feeling rules mean that male and female teachers are held to differing emotion management expectations, which in turn may restrict, by gender, the strategies culturally available to teachers (Blackmore, 2004). However, inconsistent results have been identified regarding gender effect on teacher emotion management. Some studies suggest that there are no gendered differences in how teachers manage emotions (Timms et al., 2007), while a recent review (Olson et al., 2019) revealed that female teachers use deep acting strategies, though experiencing more unpleasant emotions. Surface acting is more usually used by male teachers but with depersonalization.

In short, even though teacher emotions and their emotional labor seem to be crucial to their well-being and effectiveness and to student learning, what is missing is an understanding of the trajectory of the emotional experience (e.g., emotions and emotional labor strategies) of teachers, especially excellent teachers, across their entire career. Furthermore, as it is argued that teaching in primary schools is more emotionally intensive than other educational levels (Hargreaves, 2000; Stephanou and Oikonomou, 2018), the current study has chosen to focus on the emotional trajectory of two excellent primary teachers in China across three teacher career stages (e.g., early-, mid-, and late-career stages by Huberman, 1993), which would be explained in the result and discussion sections in detail. The following research questions are proposed:

1. What are the salient emotions that two excellent teachers have experienced at different career stages?
2. What kinds of emotional labor strategies do two excellent teachers employ at different career stages?
3. Are there any differences regarding emotions experienced and emotional labor strategies adopted at different career stages?

## Materials and Methods

The study adopts a case-study approach to explore the storied experience of two excellent teachers. The case studies of two excellent teachers will help to capture the complexity of their experiences and offer in-depth understanding of the issue derived from a real-life setting (Yin, 2017).

### Participants

The following considerations were used for the case selection. First, both participants were bestowed the Provincial Excellent Teacher Award. Second, the two participants were close to retirement (Female Alice: 57 years old; Male Frank: 58 years old) so they could retrieve emotional experiences across their entire career. Note that all participants’ names were pseudonyms. Third, the two participants came from two urban public schools in the same city. Fourth, both of them are friends of the first author as they had collaborated in different projects over the past 10 years. Life stories are very personal experiences. Trust between the participants and the researcher is very important in order to gain rich and real life stories. Fifth, both of them graduated from the Teacher-Training School (*Zhong Shi old teacher training system in China*) and gained their college qualification from the Teacher-Training College via continuing professional development.

### Data Procedure

After ethical approval, individual semi-structured interviews were utilized as a major data source, supplemented with relevant documentation and phone calls to achieve data triangulation. Each single case was examined in its entirety and included two-round individual interviews. The first-round interview lasted for about 6 h with breaks. The two participants were asked to describe the most salient emotional experiences (negative vs. positive) and emotional labor (surface acting, deep acting, and genuine expression) with background scenarios at different career stages (e.g., early, mid, and late career). The second-round interview lasted for about 3 h with breaks. The second-round interview aimed to (1) clarify any vague information from the first round of interview, phone calls, and documentation and (2) confirm the various linkages between emotions and emotional labors. The interviews were audio-recorded and digitally transcribed. Field notes and memos were made for each interview.

### Data Analysis

Thematic analysis was adopted to deal with interview data. Thematic analysis is a theoretically flexible method focusing on identifying patterned meaning across a qualitative dataset (Clarke and Braun, 2013). The data analysis procedure consisted of three stages. The first stage is the single-case analysis. In line with the first two research questions, thematic analysis was used for analyzing the first-round interview data based on emotional categories (e.g., salient positive and negative emotions) and emotional labor (e.g., three emotional labor strategies—surface acting, deep acting, and genuine expressions at the early-, mid-, and late-career stages, respectively). After analyzing the interview data, documentation, field notes, and memos for the case were examined. The second stage is the cross-case analysis of two case reports. This stage aimed to examine the similarities and differences across the two cases in response to the third research question. The third stage is to synthesize the convergent and divergent patterns. Written, informed consent was obtained from the participants for the publication of any potentially identifiable data included in this article.

## Results

The result section provides a holistic understanding of Alice’s and Frank’s emotional trajectory, respectively, with regard to their experienced emotions and emotional labor strategies adopted across three career stages.

### Case Study One: Alice’s Emotional Trajectory

#### Career Overview

Alice came from a teacher family. Becoming a teacher was not only her parents’ suggestion but also her dream in her childhood. Alice became a primary teacher at 22 years of age and has worked in two schools in the same middle-size city in China in the past 35 years. Now Alice is working at a middle-size school. She has served as the class teacher for one class since she first graduated and as Chinese subject leader but has refused to take any other leading responsibilities as she said that she has enjoyed the current working situation. Alice has taught about 12 lessons including Chinese and mathematics per week.

#### Early Career Stage

This career entry stage lasts for about 1–3 years (Huberman, 1993). As Alice described her early stage, “I was full of passion and love but being impulsive and fretful. Like a bold man, I dared to think, to speak, and to do. It seemed nothing’s gonna stop me!. I didn’t know much about the tips to manage my emotions in the beginning. On most occasions, I just expressed what I felt. You know, I was often trapped in troubles… After some unpleasant lessons, my emotions rode on a roller coaster, always with my best wishes and commitment but coming out with bad endings. I stepped back to think how to manage my emotions using tactics.”

Alice’s description showed that she experienced mixed salient emotions including love, joy, enjoyment, frustration, and disappointment in the early stage. At first, Alice was just herself all the time as she thought that love, passion, and dedication made everything. She utilized more genuine expressions regardless of positive or negative emotions for a better teaching outcome.

“I had high expectations on myself to perfect my dream profession. I remember how I taught the first year with 5-year old children. I went all out with my love and passion without doubt. When my students fulfilled the task and goals, I showed happiness and my students were happy too; When they could not, I became fretful and worried and played out these immediately. Little children are naïve but sensitive. I could see that they were scared when observing my negative expressions and reacted to me passively and scarily. Then you can image the awkward atmosphere hanging over my class.”

A similar situation to this one with the students happened also with the parents.

“In a lesson, a girl fell over on the floor and hurt her right knee with bleeding when playing with other peers. I took the girl to school clinic… when girl’s Mum came to pick up the weeping girl, I explained the details of the accident and sincerely apologized for the accident and myself. Mum was worried and annoyed. She spoke to me impolitely, “Why did you allow this happen? As a teacher, you should keep my girl safe!” I explained and apologized again and again but Mom still lost her mind. I understood her but felt aggrieved and showed it. Mum then said, “you are too young and inexperienced, I am regretful to put my girl in your class! I will ask the principal to shift my girl into another class.” I felt even more aggrieved: I did not want this to happen; it was an unexpected accident; and I tried my best to make up… The tears suddenly filled my eyes. Fortunately, my mentor came and comforted the Mum.”

After experiencing some backfiring occasions, especially witnessing the scenario in which her mentor made the girl’s Mum calm down, Alice paused to consider her strategies and figure out whether genuine expressions were enough for effectively handling the issues. Alice reported that she later tried surface acting strategies although genuine expressions were still dominant.

“As I noticed the negative effect on some occasions—sometimes driving me nuts, I deliberately suppressed or hid my negative emotions in front of my students. I realized it is very important to use different strategies to handle my emotions and try them out.”

#### Mid-Career Stage

This stage contains stabilization (4–6), experimentation (7–8), and stocktaking (9–18 years) (Huberman, 1993). Alice reported that at this stage “My emotional capacity had grown by leaps and bounds. I gained various titles and awards in my professional life at this stage. I enjoyed teaching more than before, but, of course, I still met some challenges such as new reform of teaching models which made me feel anxious and perplexed. Learning from my lessons and others, I gained a deeper understanding of handling emotional issues and, like, doing reflections. Although I still loved to express my true emotions, I was also pretty happy and tended to be skillful to employ other emotional strategies.”

From Alice’s vignettes, it appears she utilized more surface acting and genuine expression in the early phase and involved deep acting strategies later in this mid-career stage. Alice showed confidence and enjoyment more than in the early stage. This was a result of her experiments and reflections during professional practice.

“As my emotional awareness and experience increased, I found that these strategies were like a magic wand…not only hiding and suppressing, but also upgrading negative emotions could work as an effective strategy to adjust an atmosphere with students, parents, and even colleagues. By contrast, expressions of positive emotions may be not always desirable, but suppressing and hiding them on some occasions may be more effective for a better result.”

This shows that Alice indulged herself in this managing process in order to achieve better outcomes by using surface acting strategies. One more experience makes her try another strategy, deep acting, to fulfill organizational and social expectations.

“After being a class teacher for over 10 years, I became a headliner class teacher in my school and even famous in my city. I was always assigned to the top group (class) that won the successive champion year by year… My principal 1 year wanted me to take the lowest group in order to narrow the achievement gap in my school… I was reluctant as it would take lots of effort on this lowest group on track needless to say to become the top one. Another risk was that my reputation may be affected if I didn’t handle this group well… I talked about my pressure and worry with my hubby who is an important person to help me at many turning points… He said, “it may be an opportunity to bring you into a different journey, why not take it? So I took it, it did take me much effort with ups and downs of emotions, but eventually I got out of my comfort zone.”

Alice used the reframing strategy, which is a cognitive technique that helps create a different perspective for looking at a situation, person, or relationship by changing its meaning. After re-evaluation, Alice had a cognitive change. She displayed a desirable expression by modifying her evaluation on the situation, meanwhile emotions were also generated according to their expression to achieve a better result or meet workplace requirements (Yin, 2016).

#### Late-Career Stage

This stage encompasses serenity or conservatism (19–30) and disengagement (>30 years) (Huberman, 1993). Alice reported that “up to 50 s now, I am highly satisfied with myself, students, and everything. I am still taking challenges in teaching but tend to give opportunities to younger colleagues. That doesn’t mean I don’t enjoy my teaching life. In fact, my teaching life brightens up my day. This state is my most favorite one—with love, and joy, and appreciation. Although still with discordant notes, which I can easily handle or forgive using my heart and tactics… I have still expressed my true emotions and modified my emotions as I put myself in other people’s boat, but less often fake or hide my emotions on some occasions. I often feel that I am close to the essence of education.”

Alice reported more positive feelings in the final stage than in the previous two stages. Data show that Alice has achieved a harmony of body and mind more or less, which is a most desirable stage as a teacher although showing some signals of less engagement. We get a little taste of that from the following scenario.

“A boy in my class was clever but rebellious. He often teased his peers. Nothing had been changed after talking, persuading, scolding, punishing, etc. I felt very frustrated but experience made me think there should be reasons. After investigation, I understood that the boy was just jealous of his little brother who had been given much care and attention from their parents and expressed his discontent. After knowing this, I often had heart-to-heart conversations with him with my care and love… I gained his trust later and I also worked together with his parents to express our care and attention rather than scolding and separation… A good boy finally came.”

Alice tended to have more empathy to understand others and the situation at this stage. She has got more into individuals’ needs and inner worlds and focuses less on outer benefits and reputations. It is not just emotional connection but a high-order resonance between her body, soul, and profession. Just as Alice says, “most of what I do now are just spontaneous reactions and some are reactions after considerations from my heart, very comfortable.”

### Case Study Two: Frank’s Emotional Trajectory

#### Career Overview

Unlike Alice, Frank chose to become a teacher because his family was poor and the Teacher-Training School offered sufficient financial subsidy to the students so that he could support himself financially. Frank said that he was able to go to a famous university which offered a more exciting and promising career as he was the top student in his school. This background may have affected Frank’s emotional trajectory somehow. Similar to Alice, Frank became a primary teacher when he was 21 years old and has worked in the same school since then. Frank was the class teacher for 37 years and also the grade leader for about 30 years. He has taught 13 lessons including Chinese and mathematics per week.

#### Early Career Stage

Frank stated in his early stage “I tended not to stay in the teaching profession as I thought to stay with primary students was not my sincere choice for my life. I didn’t attach to my teaching spiritually but hung around with other professional opportunities… If I am asked to recall salient emotions, I remember my frustration and depression as I could not find a way out to change my life… I usually expressed what I felt regardless of negative and positive ones. Of course, I did control the frequency and intensity of my negative emotions in case my students were scared.”

From Frank’s profile, the paradox he faced at this stage was his sense of not belonging to the teaching profession. Unpleasant emotions that he experienced mainly originated from this as he was confident of handling professional issues. Despite Frank claiming not spending much energy and time on teaching, he demonstrated his talent for teaching in his early years. Frank was awarded the City Excellent Teacher in his third year. Data also showed that parents requested to send their child to Frank’s class as he was regarded as a genuine person who served as an excellent role model for their young child.

It is also clear that Frank always shared his experience and ideas with colleagues. Frank explained that his self-giving sharing and genuine expressions on most occasions may be what led to this good impression and trust from parents and colleagues. Of course, there are some discordant voices regarding interpersonal relations.

“You can’t expect that all people will like you. Some colleagues or even school leaders thought that I was not modest or even that I showed off, especially when I tried new things with my class. You know in our culture, the image is that inexperienced youngsters should highly “respect” their elders and follow after them… I adjusted my ways within my professional values to avoid any potential conflicts in this regard.”

#### Mid-Career Stage

Frank described his mid-career stage thus: “commencing from my fifth year, I gained various titles and awards and much recognition from parents, colleagues, and principals. I started to develop an interest in the teaching profession. My teaching life then was full of more enjoyment and happiness. Of course, I also felt pressure and worry but not too often after I accepted my role as a teacher and dedicated more… Compared with the first stage, I still expressed my true emotions but tried more strategies such as hide, fake, or modify my emotions.”

Frank reported that, because of his personality or habits, he still liked to express his true emotions in some situations since he believed this kind of strategy made him relaxed:

“If you see the things as simple, then they are simple. Just express what you feel. You are happy and your students are happy too. I think my students and myself had already formed a consensus. For example, innovative questions, wonderful answers, and creative interactions, all of these could touch my exciting nerve, then show it.”

Data also show that Frank often utilized surface acting strategies. He was more likely to modify the frequency and intensity of emotions to keep an emotional balance. Frank believed that teaching is a kind of art in which a teacher can perform better as their experience grows.

“Younger students are more attached to the learning atmosphere. My class was always running neutrally. If it was too hot, they would get out of hand. This would bring trouble for discipline which may affect your teaching progress. If it was too cold, they would be scared. You know, they are really good at observing your facial expressions and gestures. If I was angry, they would be very quiet and well-behaved but also it would freeze their imagination and creativity.”

#### Late-Career Stage

Frank is very famous in his city at the moment. Frank stated that “I tend to handle a butcher’s cleaver skillfully with my students and teaching life. I am really satisfied with my current stage and still keep going. I am emotionally stable although with a few emotional fluctuations but not too often… I still often express true emotions and continue the strategies used in my middle stage—say surface acting, but also try others. In most occasions, I can balance different emotional strategies and pick up the one to better fit the needs of different stakeholders.”

Frank demonstrated that he was very calm and peaceful at this stage. It was obvious that he was indifferent regardless of rain or shine:

Practice makes perfect, experience of years makes me confident with things and the environment. I can foresee the ways of things coming. I am still keen on sharing so-called “successful experiences.” Yes, there are still new challenges but not many. I ignore interferences, regardless from any parties, and do what I believe is true… I regard it as my job and I love it. However, it is not necessary to make connection between my job and heart… I am a human being first and then a teacher. I always separate my personal and professional emotions so that they do not influence each other.”

As well as surface acting and genuine expression, Frank used deep acting strategies. Frank ignored any interferences, which is refocusing, referring to the strategy by which teachers deliberately shift their attention, ignore undesirable interruptions, and focus on what they intend to do for a better outcome (Yin, 2016). Furthermore, Frank reported a disconnect between his personal and professional emotions, which is another deep acting strategy. He adjusted his role according to different situations (teacher and common person) so that his personal emotions would not have any influence on his professional activities.

## Discussion

The results provide a holistic understanding of two excellent teachers’ emotional trajectory. In this section, discussions and comparisons of the two emotional stories in the different stages are presented, followed by the effects of context and gender.

### Emotional Experiences Across Three Stages

#### Early Career Stage

The primary concern of this stage is discovering and surviving within the new arena (Huberman, 1993). A novice teacher focuses more on the task of teaching although she/he does extra and innovative activities to get with the flow. It is normally full of passion and commitment (Day and Gu, 2013). A challenge may arise regarding a sense of confidence about dealing with conflicts and boosting oneself enough to go to the next level (Fullan and Hargreaves, 2016). It is true that Alice was passionate and dedicated to her students and teaching. However, Frank did not show this characteristic but sought other career opportunities in the early stage. Alice reported mixed emotions like passion, love, and frustration, while Frank claimed to have negative emotions like frustration and depression in the early stage. Emotions like a shadow in everywhere and the teachers are doing shadowboxing. Both Alice and Frank adopted more genuine expressions supplemented by surface acting strategies. What the two teachers performed with genuine expression is described as “extroverted behavior,” demonstrating that a teacher can control the situation to gain recognition from various stakeholders (Arar and Oplatka, 2018). Gallant (2013) also found that maintaining the balance between authority and caring is identified as a major challenge for beginning teachers. This was apparently a difficult stage for both teachers, but fortunately, they finally succeeded in overcoming initial tension and worry, enabling them to carry a firm stance into the next stages.

#### Mid-Career Stage

This stage contains stabilization, experimentation, and stocktaking (Huberman, 1993). In the stabilization phase, teachers may have established a stable foundation from their previous struggles and outcomes. In the stabilization phase, teachers may try to experiment with new ideas and pedagogies for a better recognition from others. In the stocktaking phase, teachers tend to do reflections and evaluations on themselves and strive to face opportunities and challenges for the next arena. Similarly, Alice in the mid-career stage reported experiencing mixed emotions with more enjoyment but less pressure. Alice experimented with deep acting strategies although surface acting and genuine expression strategies were dominant, while Frank tended to use genuine expressions but experiment with surface acting. At this stage, both teachers deliberately removed themselves away from the emotional cycle of vulnerability and embraced a cycle of emotional containment (Blackmore, 2004), which may have led to the excellence of their careers.

#### Late-Career Stage

This stage encompasses serenity or conservatism and disengagement (Huberman, 1993). In the serenity phase, teachers may try to seek explanations of professional issues. They tend to gain positive responses from different stakeholders and be comfortable with their professional life and role. However, some teachers may shunt into the conservatism phase as they feel more rigid and stubborn and feel the lack of motivation to the profession. In the disengagement phase, teachers may lose commitment to professional activities and try to express different views of their own. Alice experienced a high level of satisfaction with everything. This may be because Alice achieved a resonance between her body, soul, and teaching in the context. Referring back to her early stage, being a teacher was her dream with sincere love so that she could build up empathy and be close to “the essence of education” as her experience grew. Likewise, Frank reported a high level of calm and satisfaction in the late stage. It is not surprising that he was very satisfied with his current state and achievement. He showed emotional stability although with a few emotional fluctuations and also kept his classroom in a similar fashion. Furthermore, Frank thought that it was not necessary to make connections between the heart and teaching profession but this did not conflict with his love and commitment to his teaching job. In the late stage, Alice used more genuine expressions and deep acting strategies but less surface acting strategies. On the contrary, Frank was inclined to adopt more surface acting and genuine expression strategies supplemented by deep acting strategies.

In short, the findings demonstrated a dynamic pattern of emotions and emotional labor, transiting from one teacher career stage to another (Arar, 2017). This is a process from “internalization” (emotion) to “externalization” (emotional labor) and from “fluctuation” to “flattening” (Hargreaves, 2005) to achieve a state of harmony or serenity. When looking at the differences of the career stages, both of them experienced the stabilization phase and quickly jumped into experimentation and stocktaking phases in the mid-career stage. Furthermore, both participants stayed in serenity mode but skipped the conservatism and disengagement phases although Alice showed a slight signal of disengagement in the late stage. The word “excellent” in the current study may account for these jumps. However, as we do not have much concrete evidence on common teachers to make comparison, future investigations are needed for clarification.

### Comparison of Emotional Experiences of Two Teachers

Examining the emotional discrepancies experienced by the two teachers, it can be seen that Alice had mixed emotions in the first stage, while Frank experienced more negative emotions. Multiple expectations by ourselves and related others may lead to anxiety, worry, and other negative consequences such as pessimism (Gallant, 2013); however, both teachers showed a great level of resilience to bounce back to a desirable professional status (Day and Gu, 2013). This finding echoes those in other studies of teachers during transition to a higher stage (Arar, 2017). Moreover, both teachers highlighted that teacher – student relations led to either positive or negative emotions. Likewise, Taxer et al. (2019) found that a quality teacher–student relationship can increase enjoyment and decrease anger. The teachers in this study also indicated that their colleagues and principals could affect their emotions and the choices of emotional labor strategies. This result aligns with other Chinese studies (Yin and Lee, 2012; Chen, 2017). Furthermore, both teachers claimed more positive emotions in the middle stage and a high level of satisfaction in the late stage, which may be a result of their reflections, confidence, and achievement.

Comparing the emotional labor strategies adopted by the two teachers, both of them employed genuine expression and surface acting strategies in the first two stages although Alice reported experimenting with deep acting strategies. More discrepancies appeared in the late stage. Alice used the combination of genuine expressions and deep acting with more empathy, while Frank adopted the combination of surface acting and genuine expressions aiming for a neutral atmosphere with an emotional balance, but both of them kept the third strategy as a supplementary one. These findings align with findings that Chinese teachers tend to undertake more complex emotional labor strategies to counteract their negative emotions (Hong and Zhang, 2019) and the expression of naturally felt emotions is seen as the most desirable one (Yin et al., 2017).

However, some interesting points are notable. First, despite both teachers adopting genuine expressions across three stages for a desirable consequence (Burić, 2019), we did not observe that they utilized more positive or negative genuine expressions which differs from the literature in which positive genuine expression tends to be adaptive, while negative genuine expression is maladaptive to the desirable outcomes (Taxer and Frenzel, 2015; Wang et al., 2019). Second, the neutral ways used by Frank could be explained by other studies (Gallant, 2013; Chen, 2019) in which teachers are encouraged to either suppress or sharpen their emotions to maintain an emotional balance. Third, Alice especially showed care and love to her students as they are the moral imperatives that “teachers hold about themselves and the work that they do” (Farouk, 2012, p. 2). Fourth, Alice exemplified using more deep acting strategies as she believed they are more effective to achieve teaching goals with emotional consonance (Yin, 2016). Although they had these differences, both teachers demonstrated how they balanced social, school, and personal demands, resulting in “a foregrounding of thoughts related to what they should or could have been doing differently” (Schutz et al., 2006, p. 345).

### Context and Gender Effect on Emotional Experiences

The findings, although indirectly, also evidence the contextual influence on teachers’ emotional expressions in the ways that they perceived their own social norms and values (Sutton and Harper, 2009; Crawford, 2018). To fulfill teaching goals and workplace requirements, both teachers intentionally modified their emotional labor strategies to avoid conflicts with different stakeholders. They demonstrated the internal oscillation that occurs as a result of the demands principal and/or colleagues place on them and the expectations they place on themselves. In Confucian culture, Chinese are expected to follow the collective norms and carry out their duties imposed by the authority (McInerney, 2008). This echoes the results from the previous studies. For example, different from the western context, Chinese teachers show negative emotions (e.g., anger) directly in order to have a desirable classroom discipline due to a relatively higher social status of the teacher in Chinese society (Schutz and Zembylas, 2009; Yin and Lee, 2012). They try to channel their emotions, body, soul, and social norms in the context as they understand their influence on their teaching or even school (Arar and Oplatka, 2018). Moreover, they tend to promote a pleasant and collective experience for a harmonious atmosphere for desirable outcomes in the Chinese context when their professional and social values are threatened (Yin, 2016). Zheng et al. (2018) identified that Chinese teachers tended to maintain public harmony or saving the faces of others, especially their supervisors, when interacting with their colleagues and principals. Therefore, emotional labor strategies are “generally a function of societal norms, occupational norms and organization norms and designate expected emotional presentations by social actors” (Hunt et al., 2008, p. 48). In other words, the choice of emotional labor strategies is affected by the social–cultural context (Mesquita and Albert, 2007).

It is observed that gender played a role in developing their emotional expressions. Alice tended to express empathy with deep acting, while Frank persisted in not linking much between teaching and heart with surface acting strategies in the late career stage. Alice tended to pursue harmony but did not segregate her personal emotions from professional ones. This could be because Alice always communicated with her husband about work. This is the classic image of feminism (Farouk, 2012). By contrast, Frank did not connect the heart with teaching and always separated his work from family matters. This does not mean that he does not love his profession but is just his way of working. This mixed finding is disparate from those in Yin’s (2016) study in which Chinese teachers believe it is better to distance personal and work emotions. The discrepancies of emotional labor strategies may be caused by gender, as Olson et al. (2019) in their recent review found that female teachers are inclined to adopt more deep acting and personalized strategies, but male teachers favor surface acting and depersonalized strategies more.

## Implications for Practice

This study offers several implications for practice in developing teachers’ emotional capacity. Firstly, it provides examples of ways for other teachers to achieve well-being and excellence as this study portrayed the emotional trajectory of excellent teachers including salient emotions experienced and emotional labor strategies adopted across three career stages. This study is a first to capture the dynamic journey of teachers and their experiences from the “excellent” perspective. Teachers’ past educational and life experiences shape their approach to the profession (Costigan, 2004), which helps unpack their professional choices at different stages. In this case, through two excellent teachers’ stories, we can understand the emotional experiences, especially the struggles and bumping moments, that lie behind the scenarios so that we can unpack the intricacies behind the profession. These retrospective and reflexive life stories help capture their career lifespan (Goodson and Sikes, 2001) from their entry into the profession to their experiences as a teacher across the profession (Mawhinney and Rinke, 2018). Therefore, this study provides useful insights for promoting teacher emotional capacity in professional practice across different career stages.

This study also provides suggestions for developing programs and interventions aiming at promoting prospective or practicing teachers’ emotional capacity. Initial teacher education programs could assist prospective teachers to foresee potential emotional tensions in their future profession and equip them with concrete emotional skills. The findings from this study that teachers experience negative emotions at the early stage especially provide a salutary image for prospective teachers. As for in-service training programs, attention could be given to analyzing the whole emotional trajectory as those already in the field need a better sense of the whole professional life. A more urgent agenda would be to provide support to teachers on building emotional capacity in order to make their teaching career more manageable and sustainable so that teachers could thrive, not just survive in their professional life (Mansfield et al., 2012).

Furthermore, the effects of gender and context identified in this study could add value to professional training. For example, the differences in choice of salient emotional labor strategies in the different career stages between Alice and Frank could help designers of professional programs give attention to gender differences. Moreover, the contextual influence (e.g., professional self, and organizational and social culture) on emotions and emotional expressions could be added into such programs. Teacher educators should be aware of being responsible for assisting prospective or practicing teachers in professional development in accordance with their needs in real situations. Indeed, all teachers should be able to engage in continuing professional development rather than just maintaining the *status quo*.

Despite offering the above implications, this study has some limitations. First, a sample of only two teachers is small; therefore, the finding may lack generalizability. Future research may engage more teachers to portray more concise patterns of emotional trajectory. Second, retrospective and reflexive life stories were used, which may be distorted as the participants may misinterpret their emotional experiences of the past. However, as real-time observation of teachers’ emotional experience across their entire career is impractical, collecting their past emotional experiences is more realistic. This is why story telling is often utilized as a common means in teacher professional development and novice teachers are encouraged to do teaching reflection (Mawhinney and Rinke, 2018). Some scholars in teacher emotion fields have provided examples such as Hargreaves’ (2000) “emotional episodes,” Erb’s (2002) “emotional incidents,” and Gallant’s (2013) “narrative emotional experiences” drawn from past experiences. Therefore, the method of life story meets the aims of the current study although it has its weaknesses.

## Data Availability Statement

The raw data supporting the conclusion of this article will be made available by the authors, without undue reservation.

## Ethics Statement

The studies involving human participants were reviewed and approved by the Education University of Hong Kong. The patients/participants provided their written informed consent to participate in this study.

## Author Contributions

JC designed the research, wrote the first draft, conducted the data collection and analysis, and did revisions. JL contributed to research design and provided the comments for the draft and revisions. JD contributed to research design, supported the data collection, provided the comments for the draft, and data interpretation and revisions.

## Conflict of Interest

The authors declare that the research was conducted in the absence of any commercial or financial relationships that could be construed as a potential conflict of interest.
